# Landscape of healthcare transition services in Canada: a multi-method environmental scan

**DOI:** 10.1186/s12913-024-11533-8

**Published:** 2024-09-27

**Authors:** Lin Li, Alice Kelen Soper, Dayle McCauley, Jan Willem Gorter, Shelley Doucet, Jon Greenaway, Alison Luke

**Affiliations:** 1 University of Toronto, Toronto, Canada; 2 Centre for Addiction and Mental Health, Toronto, Canada; 3 McMaster University, Hamilton, Canada; 4 University Medical Center Utrecht, Utrecht, Netherlands; 5 University of New Brunswick, Saint John, Canada; 6 ErinoakKids Centre for Treatment and Development, Mississauga, Canada

**Keywords:** Transition to adult care, Healthcare transition, Youth, Young adults, Environmental scan, Qualitative

## Abstract

**Background:**

Poorly supported transitions from pediatric to adult healthcare can lead to negative health outcomes for youth and their families. To better understand the current landscape of healthcare transition care across Canada, the Canadian Health Hub in Transition (the “Transition Hub”, established in 2019) identified a need to: (1) describe programs and services supporting the transition from pediatric to adult healthcare across Canada; and (2) identify strengths, barriers, and gaps affecting the provision of transition services.

**Methods:**

Our project included two iterative steps: a national survey followed by a qualitative descriptive study. Service providers were recruited from the Transition Hub and invited to complete the survey and participate in the qualitative study. The survey was used to collect program information (e.g., setting, clinical population, program components), and semi-structured interviews were used to explore providers’ perspectives on strengths, barriers, and gaps in transition services. Qualitative data were analyzed using the Framework Method.

**Results:**

Fifty-one surveys were completed, describing 48 programs (22 pediatric, 19 bridging, and 7 adult) across 9 provinces. Almost half of the surveyed programs were in Ontario (44%) and most programs were based in hospital (65%) and outpatient settings (73%). There was wide variation in the ages served, with most programs focused on specific diagnostic groups. Qualitative findings from 23 interviews with service providers were organized into five topics: (1) measuring transition success; (2) program strengths; (3) barriers and gaps; (4) strategies for improvement; and (5) drivers for change.

**Conclusions:**

While national transition guidelines exist in Canada, there is wide variation in the way young people and their families are supported. A national strategy, backed by local leadership, is essential for instigating system change toward sustainable and universally accessible support for healthcare transition in Canada.

**Supplementary Information:**

The online version contains supplementary material available at 10.1186/s12913-024-11533-8.

## Background

Internationally, increasing numbers of youth with chronic conditions and special healthcare needs are living well into adulthood and will be required to transition from pediatric to adult healthcare [1–4]. This transition is intended to be a purposeful process that includes individualized planning, coordinated transfers of care, and secure attachment to adult services [2, 5, 6]. For youth with chronic health conditions, the transition process is often fraught with many gaps and barriers, including ineffective communication and coordination; poor care continuity; inadequate access to resources; and lack of patient, caregiver, and care provider knowledge [1, 7–16]. When the transition process is not properly supported, youth can experience negative outcomes, including poor access to healthcare services, missed appointments, issues with treatment adherence, increased healthcare utilization, and health deteriorations [1, 15, 17–24]. Furthermore, research suggests that these risks may be even more pronounced for specific populations, such as youth with medical complexity [9, 20, 25, 26]. 

Despite the development of Canadian guidelines for the transition from pediatric to adult health care in 2016 [5] and a renewed call for action in 2022 [6], many jurisdictions in Canada still lack a targeted approach to address the multifaceted issues that emerge during the transition process [6]. The national guidelines developed by the Canadian Association of Paediatric Health Centres (CAPHC) [5] provide 19 person centred, clinical, and system level recommendations. These recommendations cover several areas including the need for a youth-centred approach, the development of an individualized plan, and the requirements for ongoing transition education for all groups. The Canadian Paediatric Society’s [6] call for action builds on the CAPHC guidelines with six recommendations, including a call for program funding and physician compensation for transition work, a need for collaboration between pediatric and adult healthcare providers, and a stepwise plan to increase youth autonomy (when possible) that includes flexible age cut-offs between pediatric and adult care.

The Children’s Healthcare Canada Health Hub in Transition (the “Transition Hub”) [27] is a national group of over 225 youth, caregivers, researchers, and clinicians—with coast-to-coast representation—who share the common goal of bridging the gap between current practices and evidence-based recommendations on healthcare transition [5]. We aim to accomplish this goal using a non-categorical approach (i.e., not condition specific), as much of the current research in transition is limited to specialty-specific approaches [6, 18, 28–31], despite the similarities in transition-related experiences across diagnostic and illness groups [32]. At the inaugural Transition Hub meeting in December 2019, Hub members identified that an environmental scan of Canadian transition programs was a top priority for advancing a national approach to transition. To carry out the environmental scan, we formed a multidisciplinary subcommittee of youth, caregivers, clinicians, and researchers, including the authors of this article. The aim of our project was to: (1) describe the current landscape of programs, services, and resources to support the transition from pediatric to adult healthcare across Canada; and (2) identify strengths, barriers, and gaps affecting the provision of transition services and supports in Canada.

To our knowledge, there are no published environmental scans of programs supporting the transition from pediatric to adult healthcare across Canada. Furthermore, there is a paucity of qualitative evidence on providers’ perspectives on transition that spans multiple Canadian provinces/territories and diagnostic groups [33]. Findings from this scan will support the Transition Hub’s efforts to understand the state of transition programs in Canada, as well as contribute to the development of improved resources to better support youth, families, and health and social care providers during the challenging transition process.

## Methods

Using a multimethod approach [34], we conducted this project in two phases: (1) a national survey identifying characteristics of transition programs across Canada; and (2) a qualitative descriptive [35, 36] study exploring providers’ perspectives on current strengths, barriers, and gaps affecting the provision of transition services and supports among their respective programs.

Qualitative descriptive studies can provide a comprehensive summary of an issue or event in its everyday, real-world context. As a descriptive methodology, the aim is to provide an accurate accounting of events and the meanings participants attribute to those events [37]. This type of research design is particularly useful for obtaining practical answers to questions relevant to clinicians and policy makers.

### Sample and recruitment

The sample included key informants who were involved in the delivery of programs across Canada focused on the transition from pediatric to adult healthcare. As this was an exploratory study, we broadly defined “program” to include not only distinct programs, but also other services or resources aimed at supporting transitions to adult healthcare. For both the survey and qualitative study, we aimed to recruit participants from pediatric, adult, and primary care settings across Canada.

Sample size in qualitative research can be determined by methodological norms; researcher judgment and experience; and the quality and adequacy of the data in answering the research question [36, 38]. For the qualitative study, we aimed to recruit 20–30 service providers from across Canada. Based on researcher experience and the resources available for this study, we expected that this sample size would be small enough to allow for in-depth analysis, while being large enough to address the research aims and provide a rich and novel understanding of the phenomenon [38]. 

For the survey, participants were recruited through the Canadian Transition Hub mailing list via self-referral and snowball recruitment methods. Survey participants who agreed to be contacted for a follow-up interview were then invited by email to participate in the qualitative study.

### Data collection

The survey and interview guide were developed through multiple rounds of feedback, revisions, and expert consensus by members of the Environmental Scan subcommittee of the Transition Hub, including the authors of this paper, youth/family members with lived experience, researchers, and clinicians. The survey collected program information and was administered through REDCap between October 2020 and June 2022. Between October 2021 and June 2022, a research assistant (AKS) conducted, recorded, and transcribed the interviews using Zoom videoconferencing and live transcription software. AKS then checked and de-identified the transcripts. All data collection methods (surveys and interviews) were offered in English and French.

### Data analysis

Quantitative data were analyzed using descriptive statistics, and open-ended survey questions were coded to identify transition interventions. Information about the same program reported by different participants was combined for data analysis. Qualitative interview data were analyzed in NVivo by two research team members (LL and AKS) using the Framework Method [39]. A primary analytical framework with tentative category descriptions was proposed based on the interview questions and research aims. A secondary analytical framework was developed based on the Health Care Transition Research Consortium’s Health Care Transition Model (HCT Model) [40]. We selected this model because it includes the health care system, regional agencies, and regulatory environments as important components of transition, which aligns with our primary focus of exploring strengths, barriers, and gaps in health care services. As an initial step, LL and AKS independently coded two transcripts, after which the analytic framework was revised, and then further refined throughout the analysis process (Table 1). For example, within the HCT model, we decided to focus our analysis on the environmental and healthcare system domains.

**Table 1:** Framework for qualitative analysis

Analytic Framework	Initial Codes	Final Categories/Codes
**Primary**: Interview guide and research aims	• Successful transition • Program strengths & synergies • Other facilitators • Program/provider barriers • Youth/family barriers • Gaps • Opportunities for improvement • Drivers of change • Intersection of sectors	• Measuring transition success • Program strengths • Barriers and gaps • Strategies for improvement • Drivers for change
**Secondary**: HCT Model	• Environmental domain • Health care system domain o Pediatric care o Adult or primary care • Family/social support domain • Individual domain o Adult competencies	• Environmental domain • Healthcare system domain o Organizational/ healthcare system level o Program/practice level
**Admin/Other**	• Demographic/program information • Illustrative quotes • Other	• Demographic/program information • Illustrative quotes

### Ethical considerations and rigour

For the first phase of the project (survey), our local ethics board confirmed that ethical approval was not needed to collect information on program characteristics. For the second phase of the project (qualitative study), ethical approval was obtained from the Hamilton Integrated Research Ethics Board (project #12846) in July 2021. All interview participants were provided with a link to view the participant information sheet online, and consent was obtained electronically using REDCap. While formal member checking was not done, the preliminary analysis was reviewed by the full study team, including youth, clinicians, and researchers. Their unique perspectives, assumptions, and experiences contributed to the final interpretations presented in this article. See Additional File 1 for survey questions and interview guide. Reporting of the qualitative component of this project was guided by the Standards for Reporting Qualitative Research (SRQR, Additional File 2).

## Survey results

Fifty-one participants completed the survey (English, *n* = 47; French, *n* = 4). They described 48 transition programs, services, and resources across nine Canadian provinces (Table 2). Participants were program managers/administrators, program leads, and care coordinators/patient navigators. Clinical roles included physicians, nurses, social workers, occupational therapists, pediatric dentists, and a dietician and family counsellor (Table 3). Of the 48 programs, 39 (81%) reported having dedicated personnel to support transition, and 16 (33%) had been evaluated. There was a wide range of transition interventions that focused on: (1) transition processes and care continuity; (2) self-management; (3) life skills, autonomy, and coping; and (4) care delivery approach.

**Table 2:** Program Location by Province

Province	48	%
Ontario	21	43.8%
Québec	6	12.5%
Alberta	6	12.5%
British Columbia	5	10.4%
Manitoba	4	8.3%
New Brunswick	2	4.2%
Nova Scotia	2	4.2%
Newfoundland	1	2.1%
Saskatchewan	1	2.1%

**Table 3:** Participant roles

Role	51	%
Program lead	14	27.5%
Care coordinator/patient navigator	9	17.6%
Physician	9	17.7%
Nurse	7	13.7%
Program manager/administrator	6	11.8%
Social Worker	4	7.8%
Occupational Therapist	3	5.9%
Dentist	2	3.9%
Other	2	3.9%

Other included a dietitian and family counsellor. Some participants identified with more than one role.

### Transition processes and care continuity

Transition interventions were often aimed at preparing youth and their families for transition by creating a better understanding of transition processes. Programs offered general information, tools, and resources on transition, as well as individualized navigation support and consultation for youth, parents, and providers. Written transition plans and medical summaries (e.g., the 3-sentence summary) were a means of supporting care continuity by facilitating information transfer to adult and primary care providers.

### Self-management

Management of physical and mental health involved increasing knowledge about specific medical conditions and building skills to self-manage symptoms and disease activity. Skills in self-management included how to refill prescriptions, book appointments, communicate with adult providers, and self-advocacy. Self-management skills and transition readiness were frequently assessed using the Transition Q [41] and ON TRAC [42] questionnaires.

### Life skills, autonomy, & coping

Life skills were related to activities of daily living, social life, and recreation, as well as employment and post-secondary education. These skills were discussed and developed through education webinars, workshops, youth mentorship, and coaching approaches. Programs also focused on mental well-being and provision of psychological support from social workers, psychologists, and through peer-support groups.

### Care delivery approach

Lastly, some programs adopted care delivery approaches specifically aimed at supporting transitional-aged youth, including youth-focused or combined pediatric-adult clinics and programs. Table 4 provides a summary of the frequency, by program, that each transition intervention was reported in the open-ended survey responses. Specific program details, including clinical population, setting, and program description are provided in Table 5.

**Table 4:** Transition interventions reported by Programs

Focus	Transition Intervention	Programs Reported (*N* = 48)
Transition processes & care continuity	General information, tools, and resources related to transition	17 (35.4%)
	Individualized navigation/care coordination	13 (27.1%)
	Standardization and quality improvement (frameworks, policies, clinical pathways, guidelines, etc.)	10 (20.8%)
	Written transition plan and/or medical summary	8 (16.7%)
Self-management	Self-management and self-advocacy education and support	16 (33.3%)
	Transition readiness assessment	8 (16.7%)
Life skills, autonomy, & coping	Life skills and goal setting	13 (27.1%)
	Psychological/peer support	11 (22.9%)
	Education and/or employment	10 (20.8%)
Care delivery approach	Youth-focused clinic/program	16 (33.3%)
	Combined pediatric-adult clinic and/or overlap in care	15 (31.3%)

Note: The interventions in this table were extracted from open-ended survey responses, which varied in their level of detail. One program did not provide enough details about specific transition interventions to be counted in this table

**Table 5:** Program characteristics. Programs are categorized into three groups: (1) Pediatric programs: only provide services up to the typical age of transfer (18 or 19 years, depending on jurisdiction); (2) bridging programs: provide services that span the typical age of transfer (includes both pediatric and adult healthcare institutions); (3) adult programs: only provide services after the typical age of transfer

Organization, Program (Province)	Age range	Setting; clinical population	Description of Program
Pediatric (*N* = 22)			
Alberta Children’s Hospital, Adolescent Transition Clinic (Alberta)	14–18 years	Hospital/outpatient, virtual; physical, mental, and developmental conditions	Offers medical knowledge and resources to support general life skills. Provides transition resources and generates individual transition plan
Alberta Children’s Hospital, Well on Your Way Program (Alberta)	13–18 years	Hospital/outpatient; physical and developmental conditions	Provides tools and resources with information about the transition process. Provides navigation support for patients, their families, and healthcare teams
Glenrose Rehabilitation Hospital (GRH), Pediatric to Adult Transition Project (Alberta)	12–18 years	Hospital/outpatient; physical and developmental conditions	Transition framework includes transition domains, guidelines,, roles and responsibilities, equity access, transition tools, caregiver and patient workshops and linking to community supports.
Stollery, Pediatric Diabetes Education Centre, Adolescent Transition Program (Alberta)	16–18 years	Hospital/outpatient; physical conditions (diabetes)	Education on transition topics and teen-focused appointments
BC Children’s Hospital, Cystic Fibrosis Clinic (British Columbia)	12–18 years	Hospital/outpatient; physical conditions (cystic fibrosis)	Services based on the On TRAC transition model consisting of transition clinical pathways, readiness measures, and joint clinics for patient handover.
BC Children’s Hospital, Division of Adolescent Health and Medicine (British Columbia)	17–19 years	Hospital/outpatient, community-based; physical, mental, and developmental conditions	Development of transition resources (e.g., On TRAC); provide educational materials to support the transition process.
British Columbia Pediatric Society	N/A	Community-based, for consulting Pediatricians	Resources for pediatricians and their patients/families to support transition.
Health Sciences Centre, Children’s Heart Centre (Manitoba)	Up to 18 years	Hospital/outpatient; physical conditions (cardiology)	Combined transition clinic; information on transition process and adult clinic; condition self-management through teaching sessions with clinicians.
IWK Health, You’re in Charge (Nova Scotia)	13–18 years	Hospital-based, primary care, virtual; physical, mental, and developmental conditions	Focus on chronic condition self-management.
Children’s Hospital of Eastern Ontario (CHEO), Chronic Pain Service (Ontario)	16–18 years	Outpatient, virtual; physical, mental, and developmental conditions (chronic pain)	Education on personal health, how to refill prescriptions, book appointments, and self-advocate. Joint meetings with pediatric and adult providers; 6-week psychoeducational groups
ErinoakKids (Ontario)	12–19 years	Community-based	Support transition to adult service system and identify appropriate post-secondary education opportunities; long-term planning and resources to support personal goals
Hamilton Health Sciences (McMaster Children’s Hospital), Diabetes (Ontario)	17–18 years	Hospital/outpatient; physical conditions (diabetes)	Assess self-management, introduce differences in adult healthcare, address risk-taking behaviours and mental health.
Hamilton Health Sciences (McMaster Children’s Hospital), My Transition (Ontario)	12–18 years	Hospital/outpatient, virtual; developmental conditions	MyTransition hospital-wide program includes tools, Transition Q, 3-sentence summary, and graduation certificates at the last clinic visit.
Hamilton Health Sciences (McMaster Children’s Hospital), Neurology (Ontario)	Up to 18 years (upon referral to adult services)	Hospital/outpatient, virtual; physical, mental, and developmental conditions (neurology)	Provides overview of available transition resources,
Hamilton Health Sciences (McMaster Children’s Hospital), Teen Transition Clinic (Ontario)	12–18 years	Hospital/outpatient, virtual; developmental conditions	Focus on self-management skills and work with families of youth; dedicated teen-transition clinic.
Hamilton Health Sciences (Ron Joyce Children’s Health Centre), Children’s Developmental Rehabilitation Program (Ontario)	Up to 18 years	Outpatient; physical and developmental conditions	Transition planning activities, skill-building sessions related to transition topics and general life skills, clinical consultation with families/patients, provision of transition resources; teen-transition clinic
London Health Sciences Centre (Children’s Hospital of Western Ontario), Department of Pediatrics (Ontario)	12–18 years	Hospital/outpatient; physical and developmental conditions (neurology, GI, and hematology)	Assess transition readiness and provide support from a transition navigator.; offer transition clinics to adolescent patients.
London Health Sciences Centre (Children’s Hospital of Western Ontario), Pediatric Diabetes (Ontario)	15–18 years	Hospital/outpatient, virtual; physical, mental, and developmental conditions (diabetes)	TRAC Transition readiness assessment and complication clinic to discuss transition and adolescent related materials.
London Health Sciences Centre (Children’s Hospital of Western Ontario), Pediatric Neurology (Ontario)	16–17 years	Hospital/outpatient; physical, mental, and developmental conditions (neurology)	Transition clinic with joint meetings with pediatric and adult providers.
SickKids, Cardiology Transition Program (Ontario)	14–17 years	Hospital-based; physical conditions (cardiology)	Provides education on cardiac knowledge, self-management, self-advocacy skills.
CHU Sainte-Justine, Médecine de l’Adolescence (Québec)	12–17 years	Hospital/outpatient, community-based; mental health	In addition to physical and mental health, focus on social life, education, recreation, and sexual health; medical summary provided by pediatric team
Jim Pattison Children’s Hospital, Pediatric Nephrology (Saskatchewan)	15–18 years	Hospital/outpatient, inpatient, virtual; physical and mental conditions (nephrology)	Provides education on medical and dietary self-management, sexual health, education and career planning, and mental and physical health; joint visits with adult and pediatric providers
Bridging Pediatric to Adult (*N* = 19)			
Alberta Health Services, Emerging Adult Treatment Clinic (Alberta)	16–24 years	Outpatient; virtual; mental health conditions	Provides mental health and general life skills (e.g., education, relationships, and employment) support.
IBD Centre of BC, Young Adult IBD Clinic (British Columbia)	16–25 + years	Outpatient; physical conditions (IBD)	Multidisciplinary program with psychologist, dietician, and GI support.
Rehabilitation Centre for Children, Adolescent Clinic (Manitoba)	Up to 21 years	Outpatient; physical and developmental conditions	Youth and young adult clinic focused on orthopedic needs of youth who use assistive technology.
St. Boniface Hospital, Manitoba Adult Congenital Heart Clinic (Manitoba)	16–20 years	Hospital-based; physical conditions (cardiology)	Support for coordination of transfer of care. Transition clinic run with the Variety Children’s Heart Centre.
Winnipeg Health Sciences Center, Pediatric Diabetes (Manitoba)	16–25 years	Outpatient; physical conditions (diabetes)	Transition coordination to adult diabetes care with phone, virtual, or in-person support.
Ability New Brunswick, Transition New Brunswick (New Brunswick)	16–30 years	Community-based; physical conditions (mobility focused)	Ensure youth have access to equal opportunities for education and employment; identify strengths, goals, and resources
University of New Brunswick, NaviCare/SoinsNavi (New Brunswick)	Up to 25 years	Community-based, virtual; physical, mental, and developmental conditions	Coordinate patient care, facilitate transitions and connect families with resources.
IWK Health, Children’s Health Program (Nova Scotia)	Up to 25 years	Hospital-based; physical, mental, and developmental conditions	Individual clinics provide transition support by way of preparing families for transition at time of diagnosis, assess transition readiness, involve adult specialists in transfer or joint clinics.
Hamilton Health Sciences, Pediatric and Adult Rheumatology Transition Clinics (Ontario)	14–22 years	Hospital/outpatient, virtual; physical conditions (rheumatology)	Combined pediatric/adult rheumatology clinic and young adult clinic, complete Transition Q and goal setting with allied healthcare provider.
Holland Bloorview Kids Rehabilitation Hospital, Transition Strategy (Ontario)	Up to 21 years	Inpatient, outpatient, community-based, virtual; physical and developmental conditions	Involves life skills programs, hired Youth Facilitators on healthcare team, Friendship and Belonging virtual ‘hangouts’, ‘Getting Started Early’ webinars, Youth Mentors/Leaders are embedded throughout program.
McMaster University, Adult/Pediatrics GI Joint IBD Transitions Program (Ontario)	16–22 years	Hospital-based; physical conditions (IBD)	Support adolescents to gain skills to transition
Parkwood Institute & Western University, Transitional and Lifelong Care (TLC) Program (Ontario)	14 + years	Hospital/outpatient, virtual; physical and developmental conditions	Review of transition plans, address concerns, and make referrals to social work and occupational therapy.
Pediatric Oral Health and Dentistry (Ontario)	12–25 years	Primary care, community-based; mental and developmental conditions (oral health)	Creating a series of transitions forms for dentistry based on the GOT Transition.
SickKids & University Health Network, Hematology (Ontario)	Up to 20 years	Hospital/outpatient, virtual; mental, physical and developmental conditions (sickle cell disease and thalassemia)	Monthly transition clinic, disease knowledge assessment and education, goal-setting, support to navigate post-secondary education and employment, and healthy living strategies. Offer group workshops and education sessions, system/resource navigation, quality improvement, and partnering with community patient organizations
Women’s College Hospital, Toronto Academic Pain Medicine Institute, Young Adult Clinic (YAC) (Ontario)	17–25 years	Hospital/outpatient, virtual; mental and physical conditions (chronic pain)	Offers interdisciplinary assessment and treatment at young adult clinic.
CHU Sainte-Justine, Programme Parachute (Québec)	14–21 years	Hospital-based; physical, mental, and developmental conditions (diabetes, neurology, and cardiology)	Facilitates transition and focus on building autonomy and self-management in planning and organizing care; building a program for standardized transition care
Lethbridge-Layton-Mackay Rehabilitation Centre, TranXition Program (Québec)	15–25 years	Outpatient, community-based, virtual; physical conditions	Coaching approach to guide independence, autonomy, self-determination, goal attainment, physical and mental wellbeing; rehabilitation services to youth; general life skills support and offer out-patient services through peer groups
Lethbridge-Layton-Mackay Rehabilitation Centre, Youth and Young Adult Program (Québec)	8–24 years	Outpatient; physical conditions	Transition-focused planning meetings and transition support from specialized educator and social worker.
McGill University, Oral Health Clinic for Neurodevelopmental Diversity (Québec)	14 + years	Hospital/outpatient, virtual; developmental conditions (oral health)	Provide full range of dental services and virtual visits as desensitization or diet and oral hygiene advice.
Adult (*N* = 7)			
Alberta Health Services, Young Adult Neurorehabilitation Clinic (Alberta)	18–25 years	Outpatient, virtual; physical, mental, and developmental conditions (neurology)	Spasticity management, general transition support, referrals to psychologists and social workers.
Providence Health Care, Scotiabank Youth Transition Program (British Columbia)	18–24 years	Hospital/outpatient; physical, mental, and developmental conditions	Development of initiatives to improve care coordination, access to resources on transition, host workshops, and build peer support.
Memorial University, Forest Road Clinic (Newfoundland and Labrador)	18 + years	Primary care, community-based; physical, mental, and developmental conditions	Initiates and follow-up on referrals to specialists and allied health professionals in the adult system; provides comprehensive package to family physicians
Hamilton Health Sciences, Michael G. DeGroote Pain Clinic (Ontario)	18 + years	Hospital/outpatient; physical conditions (chronic pain)	Provides transition assessments 2-months prior to transfer at 18 years. Focus on transition needs/goals with a Young Adult Support Worker. Pain-management group with focus on building independence. Quarterly phone call to all young adults up to 25 years.
London Health Sciences Centre, Adult Cystic Fibrosis Program (Ontario)	18 + years	Hospital-based; physical conditions (cystic fibrosis)	Formal education program that provides knowledge of disease, self-management, available resources, mental and physical health.
McMaster University, Division of Nephrology, Transitional Clinic (TLC) (Ontario)	18–25 years	Hospital/outpatient; physical conditions (nephrology)	Support from care coordinator, bridging visit attended by pediatric team, utilize Transition Q, support from social work and psychologist.
l’Institut de Réadaptation en Déficience Physique de Québec (IRDPQ) (Québec)	18–21 years	Outpatient; physical conditions (neuromuscular, amputees, spasticity and myelopathies)	Pediatric summary file and initial assessment by the adult team, personalized according to individual needs.

## Qualitative findings

Twenty-three transition providers from 22 unique programs, 19 organizations, and 6 provinces completed interviews. While both French and English interviews were offered, all interviews were completed in English. Qualitative findings were organized into the following five topics: (1) “measuring transition success” encompasses participants’ views on what defines a good transition and how transition success should be evaluated; (2) “program strengths” describes what is currently working well to support transition within the participants’ programs; (3) “barriers and gaps” includes barriers that providers face in supporting transition, as well as unmet service needs faced by youth and families during transition; (4) “strategies for improvement” entails concrete actions or interventions that have the potential to improve current transition services; and lastly, (5) “drivers for change” examines the underlying mechanisms that are needed to spur more widespread changes to support transition.

### Measuring transition success

Participants emphasized the importance of evaluating transition success, which was seen as valuable for both assessing youth readiness for transition, as well as monitoring program effectiveness. Participants described indicators of successful transition ranging from clinically relevant or health-related outcomes (e.g., care continuity, care satisfaction, self-management skills and knowledge, indicators of physical and mental health) to broader themes of developing adult competencies (e.g., general life skills, residential autonomy, independence, education, employment). For example, Participant 19 spoke of measuring transition success through clinical indicators:

*“I think a large component of it is patient satisfaction and confidence throughout the process. So that they know who to call. They don’t feel lost. They’re not confused. They never have a gap in time*,* where they have no one who’s kind of taking care of them.”*

On the other hand, Participant 15 emphasized non-clinical elements:

*“For me for successful is beyond the medical—it’s looking at all aspects.”*

Participants also noted that transition is gradual and should be measured by both process and outcome indicators. Participant 1 shared that *“there’s no one size fits all. But definitely*,* we would like to see progress in kind of a minimum level of*,* you know*,* acquisition of knowledge and self-care skills.”* Setting and meeting individualized goals over time can also show progression towards transitioning to the adult system, and this progress should be captured in measures of transition success. Overall, a successful transition is multifaceted and should be evaluated using a holistic and lifespan approach.

### Program strengths

At the practice level, some participants described the advantages of having a collaborative, intrinsically motivated team to share transition-related responsibilities within their program. Others spoke of the importance of having a designated person, such as a transition coordinator, dedicated to supporting youth and families. Transition planning with youth and families was facilitated by offering flexible options for care delivery and communication, as described by Participant 17:

*“Our groups run at the time that are best for the youth*,* including weekends…We’ve also switched our communication modes with them*,* because they don’t do emails*,* or very little. They do messenger or Instagram.”*

Specific program features were also viewed as strengths, such as care delivery models focused on overlap of pediatric/adult services: *“I think doing our combined clinic has been helpful because they put a face to a name. They meet somebody from the adult side and that certainly makes them a lot more comfortable”* (Participant 23). Strengths-based approaches to transition planning and goal-setting, and additional time during appointments to address non-health related concerns were also commonly endorsed program strengths.

At the organizational and healthcare system level, some participants attributed the success and sustainability of their programs to transition being valued and supported across all levels of the organization: “*We have very strong support from all levels of management”* (Participant 8). Institutional or government resources for transition services was also viewed as essential, as was organizational support for meaningful family and youth leadership in the design of transition services:

*“We have a really strong partnership with all of our family leaders and our youth leaders*,* and they help inform us every step of the way”* (Participant 14).

At the environmental level, all participants acknowledged the value of cross-sectoral collaboration to address the multifaceted needs of youth and families during transition. For example, Participant 17 shared that “*linking up and partnering up with a variety of community organizations”* was working well in their program. In particular, the potential for synergistic partnerships between the health and education systems was highlighted:

*“We work together collaboratively to do a post-secondary program for students with physical disabilities who are making that leap into college”* (Participant 11).

### Barriers and gaps

At the practice level, a common barrier was the lack of engagement and awareness among healthcare providers of the importance of transition and the availability of existing transition supports. Participant 10 shared that *“one of the things that we’d like to do is to see if we can engage some of our adult partners or to do some sort of community partners sort of piece…but we haven’t been able to get any traction on that.”* Participants also reported that healthcare providers often did not feel prepared to support non-health-based needs, such as employment and education, or lacked training in caring for adults with complex healthcare needs:

*“I think there’s a lot of gaps in terms of knowledge and how to appropriately care for these youth”* (Participant 16).

At the healthcare system level, lack of resources, in the form of funding, staffing, or time, was frequently reported as a barrier. Resource constraints made it difficult for providers to dedicate time towards transition and sustain services long-term. Remuneration structures were a barrier when providers were not compensated for the additional time required for providing transition support. For example, Participant 18 described how inconsistent funding has led to a lack of continuity in transition services over the years:

*They develop a program*,* which our hospital has done a couple times*,* and they support a year of transition care. And then*,* the funding is over at the end of the year*,* and everything just goes away. And then two years later*,* maybe we’ll try to pick it up*,* but it’s with a new person. So*,* there’s not a lot of continuity*,* and I find that is a huge barrier for our kids.*

Additionally, the use of multiple models of service delivery across different programs can pose challenges to implementing organization-wide standard measures or guidelines, highlighting the need for frameworks that can be tailored to different populations and care contexts. Specific gaps in services for transition-aged youth included: insufficient social work and mental health services; decreased availability of occupational therapy and physiotherapy in the adult system; waitlists for adult services resulting in care delays; and lack of a centralized platform for families to find information.

Environmental and societal factors related to accessibility and equity also posed barriers to providing transition support. These factors included burdensome travel requirements to access services, lack of access to or poor engagement in virtual care, and language barriers, as voiced by these participants: *“many*,* many*,* many of our families live in remote areas”* (Participants 7), and *“most of [our patients] are refugees or immigrants—about 98% of them don’t speak English”* (Participant 18). Participants also shared that equity-deserving groups, such as youth with developmental disabilities or medical complexity, experience the greatest gaps in services and are often underrepresented in clinical and research initiatives:

*“I have a large amount of youth that have developmental disabilities*,* so a large portion of them can access technology*,* but then there’s also that portion that can’t access technology*,* at least independently”* (Participant 7).

### Strategies for improvement

Within the healthcare system, the most frequently suggested strategy was to employ dedicated personnel focused on transition (e.g., transition coordinators), particularly in the adult system: *“I feel like we’re missing some players*,* maybe*,* in the adult world as far as a coordination type*,* or coordinator role”* (Participant 9). Strategies focused on care delivery included: embedding and standardizing transition practices across an organization; expanding existing transition programs; providing overlap between pediatric and adult care; implementing policies to support flexibility in the age of transfer; streamlining care logistics (e.g., referrals); and tailoring services to youth preferences. For example, Participant 1 advocated for transition to be *“something that is embedded in anyone who is looking after a youth with a chronic condition in any way*,*”* while Participant 3 shared feedback from their patients on the value of joint pediatric-adult appointments:

*“We’ve heard from patients that what would be a really nice kind of way to do the handover is to have their final appointment with their pediatric team be their first appointment with us [(the adult team)].”*

In the environmental domain, participants suggested building partnerships and communities of practice, such as the Transition Hub, focused on *“bringing [resources and knowledge] together and making it accessible to one another”* (Participant 3). Not only do these networks allow youth, caregivers, researchers, and clinicians to exchange resources and lessons learned and work collaboratively toward common goals, but the sense of community also helps individual providers feel more connected and motivated to implement local changes. Education and training—for youth, families, and providers—to increase awareness of transition resources and processes was also highly endorsed. Lastly, a centralized platform for families and providers to connect and find information related to transition (e.g., employment, education, housing/independent living, healthcare) was seen as an essential, but missing, resource: *“We don’t have yet a centralized place where parents can find their information or youth can find their information or connections that are readily made between a network of various actors working in transition”* (Participant 17).

### Drivers for change

In addition to having feasible, concrete strategies for improvement, it was reported to be essential to have the motivation and drive for change. The most common drivers for change identified by participants were youth and family stories and motivated transition champions, as highlighted by the following two participants:

*“I think families are often a driver of change*,* which made my centre and my director pay attention because…when we did a family survey…[transition] was one of the top five areas of interest identified by families.”* (Participant 9).

*“The reality is that most of the practice changes that I have seen in transition*,* come from champions*,* so there have to be sort of people in the system*,* who recognize that this is a problem and take it on and run with it and advocate for it.”* (Participant 19).

These messages emphasize the critical role of collaborative advocacy in securing resources for transition.

Research and quality improvement were also reported as essential for inspiring and justifying change. Participants spoke of the need for program evaluation and consensus on quality indicators for transition, including longitudinal, quantitative data to evaluate the long-term effectiveness of transition services and programs. Participants felt that more research on evaluating transition could provide powerful evidence for funding bodies to support the expansion of transition services.

Beyond factors that intrinsically motivate providers to change their practice, participants emphasized the need for external drivers executed through healthcare policy. External drivers that were identified include provincial or national standards of care, funding and resources for transition, hospital accreditation criteria, and financial incentives for providers and healthcare organizations. Participant 1 stressed the importance of *“institution-wide support…that values and legitimizes transition as a standard of care*,*”* and suggested that transition *“should be part of the hospital accreditation process.”* Meanwhile, Participant 10 reflected that *“there is probably a piece of revenue generation*,* especially among family physicians in terms of how do you support them to be able to bill for…transition.”* Furthermore, some participants believed that pressure from broader society is needed, and they emphasized the importance of shifting societal perceptions of disability and framing support for transition as an *“important societal need and benefit as part of preventative health care*” (Participant 3).

## Discussion

We describe the current landscape of Canadian programs, services, and resources to support young people in their transition to adult healthcare. Whereas Grant and Pan previously compared five transition programs across Canada [43], our survey, which reports on 48 transition programs from nine Canadian provinces, reflects the significant expansion of Canadian transition services and programs over the past decade. Additionally, while Splane and colleagues previously explored providers’ experiences of healthcare transition in New Brunswick and Nova Scotia [33], other Canadian studies have been limited to a single province. To our knowledge, this environmental scan is the first study to examine providers’ perspectives (*n* = 23) on transition across Canada. We found that most of the surveyed programs are based in hospital (*n* = 31/48, 65%) and outpatient (*n* = 35/48, 73%) settings. The qualitative findings offer insights on service providers’ perspectives regarding: what constitutes a successful transition, program strengths and barriers, concrete strategies for improvement; and underlying mechanisms for system change.

Notably, some ideas surfaced under more than one topic. For example, some participants described dedicated personnel, flexible care delivery, and overlaps in care as “strengths” of their individual programs, while others voiced similar ideas as “strategies” that they were either working toward or advocating to be implemented on a larger scale. Likewise, participants suggested several “drivers for change” that reduce “barriers” and support “strategies” that were identified (e.g., national standards to improve provider awareness; financial incentives to address funding barriers and support dedicated personnel). We draw attention to these recurrent themes; the repeated emphasis gives credence to their potential as targets for future interventions and policies to support healthcare transition in Canada.

### Accessibility of transition services

Increasing access to transition support is a pressing issue for healthcare systems [2, 6, 44–46]. Structural factors limiting healthcare access were identified as key barriers to providing transition support in this study, including extensive travel requirements, poor access to virtual care, language barriers, and limited healthcare funding and staffing. These continue to be ubiquitous issues within the Canadian healthcare system, and resource limitations have been reported as prominent barriers in other Canadian studies exploring providers’ perspectives on transition [33, 47, 48]. Findings also revealed that equity-deserving groups tend to face the most difficult transitions and experience the greatest gaps in services, which is consistent with other published literature [6, 9, 44, 45, 49, 50]. Furthermore, as most of the surveyed programs were condition-specific and lacked consistency in ages and populations served, gaps in services continue to exist for those who do not fit into specific program parameters, such as youth with rare diseases [51]. 

These findings underscore the importance of embedding transition into the practice of all providers who care for adolescents and young adults, including pediatric, adult, and primary care providers [5, 44, 52]. To support more universal access to transition support, transition interventions (e.g., transition preparation, system navigation, care models that ensure continuity) should be viewed as essential, not auxiliary, components of care. This shift in provider attitudes toward transition will help to reinforce strengths (e.g., transition being valued) and mitigate barriers (e.g., lack of provider engagement) identified in this study.

### Local strategies to improve transition

While barriers and enablers of successful transitions have been widely discussed in the literature [1, 11, 13, 15, 25, 53, 54], our study reports novel findings on provider-identified, actionable strategies for improving transition, as well as drivers for practice change. The most commonly suggested strategy by participants in this study was to ensure the availability of transition coordinators or dedicated personnel to support transitions, echoing recent recommendations from care providers in Atlantic Canada [33]. Although most of the surveyed programs (*n* = 39/48, 81%) had dedicated personnel to support transition, qualitative findings revealed that relying on a single person to provide transition services could potentially lead to issues with sustainability. While the issue of long-term program sustainability has previously been raised in the literature [54], it has yet to be addressed through systemic solutions. Our qualitative findings emphasized the importance of organizational support for ensuring long-term sustainability of transition services and programs. Healthcare organizations can drive the uptake of existing recommendations and guidelines for healthcare transition through the implementation of organizational policies and standards, personnel support for transition, and mechanisms that enable tracking of transition interventions and outcomes (e.g., through electronic medical records).

### A national approach to amplify practice change

Beyond individual organizations, more widespread practice changes are needed [55]. Patient, caregiver, and provider champions are essential for inspiring these changes. Furthermore, while intrinsic motivation can spark local initiatives, extrinsic motivators are needed to drive change at a system level [56]. These external drivers can include provincial or national standards for transition care [5, 52, 57], system-wide policies that promote flexibility (e.g., in the age limit for pediatric care) [6, 57, 58], transition indicators [59, 60] embedded into healthcare accreditation criteria to measure success and benchmarking, and financial incentives for providers and organizations to improve transition processes and better support youth transitioning to adult care [45, 61]. 

A national approach is critical to overcome systemic barriers and scale the current state of Canadian transition services beyond pilot and local programs and toward long-term sustainability and universal care access. To facilitate access to care, we propose that a next step is to establish a central platform maintained by the Transition Hub. The platform would provide up-to-date information on transition programs, services, and resources in Canada. The need for a central directory has frequently been identified in this study, as well as anecdotally: we as active members of the Canadian Health Hub in Transition are often consulted by researchers and clinicians, both internal and external to the Hub, to share information on transition programs to help guide the design and delivery of transition services. Findings from this study will also inform future directions for the Transition Hub, such as: (i) increasing uptake of national transition guidelines; [5] (ii) fostering synergistic collaborations to accelerate the spread and upscaling of practice innovations; and (iii) partnering with adult and primary care providers to advance a unified vision of transition care in Canada. Importantly, future efforts to improve the accessibility and quality of transition services should be made in partnership with youth and caregivers, including seeking their perspectives on service design and delivery.

### Limitations

A limitation of this environmental scan is our reliance on the membership of the Transition Hub for recruitment. Although the Hub boasts over 225 members who are invested in healthcare transition issues (both professional and personal), with strong pan-Canadian representation, we acknowledge that there are transition programs not represented in the Hub that our recruitment efforts missed. However, a key strength is that we offered data collection in both English and French, effectively increasing the scope of our recruitment strategy. An additional limitation is that we did not elicit youth/family perspectives as part of data collection, thereby limiting the scope of the study findings. This limitation should be considered when interpreting implications for patients and families. While youth/family perspectives were not included in the data, the Environmental Scan subcommittee includes both youth and family members, who have been involved throughout the research process, including data analysis and interpretation.

## Conclusions

Our study sheds light on the diverse landscape of Canadian transition programs, services, and resources, drawing from the insights of transition service providers from nine Canadian provinces. Findings underscore the multifaceted nature of successful transitions, highlight program strengths at various levels, and identify key barriers impacting healthcare access. Actionable strategies and drivers for change offer a roadmap for improvement, emphasizing the need for a national approach, supported by local leadership, to ensure sustainable, universally accessible transition support in Canada.

## Supplementary Information

Supplementary Material 1:  Survey Questions and Interview Guide.  Survey questions and semi-structured interview guide

Supplementary Material 2:  Standards for Reporting Qualitative Research (SRQR) Checklist.  SRQR Checklist indicating which items were reported and where in the article they can be found

## Data Availability

The data from the current study are available from the corresponding author on reasonable request.
