# Supplementary material for: Landscape of healthcare transition services in Canada: a multi-method environmental scan
Source: BMC Health Serv Res. 2024 Sep 27;24:1114. doi: 10.1186/s12913-024-11533-8 (PMC11428857; doi:10.1186/s12913-024-11533-8)
Supplement: Supplementary file 1 — Supplementary Material 1: Survey Questions and Interview Guide. Survey questions and semi-structured interview guide [file 12913_2024_11533_MOESM1_ESM.docx]

**Additional File 1: Survey Questions and Interview Guide**

**Survey Questions**

1. What is the name of your organization? [open text box]
2. What is the name of the service or program providing healthcare transition services/support? [open text box]
3. What is your position within the program? [open text box]
4. What population does the program serve? Check all that apply.
   1. Physical conditions
   2. Mental conditions
   3. Developmental conditions
   4. Other
5. Please provide details about the population served (e.g. specific conditions, geographical locations, newcomers to Canada, Indigenous groups, healthcare providers, etc.): [optional; open text box]
6. What age range of patients does the transition service or program serve? [open text box]
7. What is the setting of the program? Check all that apply.
   1. Hospital based
   2. Inpatient program/service
   3. Outpatient program/service
   4. Community based
   5. Primary care
   6. Virtual
   7. Other. Please specify: [open text box]
8. Is there a dedicated person or team who focuses on transition? [Yes/No]
   1. What are their titles and roles (including health professional disciplines, if applicable)? [optional; open text box]
      1. Please explain: [optional; open text box]
9. Please provide a brief summary of the services that your program provides for youth transitioning to adult healthcare. (Please provide any details that you think are relevant. For example: What are the components of the program? What are the hours of availability? Is the program virtual or face-to-face? How many clients do you serve in a year?) [open text box]
10. Has the transition program/service (or transition process) been evaluated? [Yes/No]
    1. If yes, briefly describe how the transition program/service or process has been evaluated. For example, what measures or feedback were included in your evaluation: patient satisfaction surveys, disease indicators, service utilization, transition readiness, adherence, etc. [open text box]
    2. Has this been published, either as peer-reviewed or grey literature (not peer-reviewed)? [Yes/No]
       1. Please share a citation of the published material, or upload a copy in the field below. [open text box/file upload]
11. If you know of other pediatric to adult healthcare transition programs, please provide us with the contact information (name, email, website) of someone we could talk to. [optional; open text box]
12. Can we contact you within the next 6 months to discuss further details about the program if needed? [Yes/No]

**Semi-Structured Interview Guide**

**Interview Questions (sub-questions are prompts):**

1. Can you tell me about how your program or service supports the transition from pediatric to adult health care?
   1. What is the name of your program?
   2. (If not clear from the survey: can you describe the setting and location of your program, and what geographical areas they service?)
   3. What is your discipline/role within the program?
   4. Did staff in your program receive any training related to health care transition? What did that look like?
   5. How often is the program/service delivered (daily, monthly, quarterly, etc.)?
   6. How many clients/patients does your program or service see in a year?
   7. Are there any transition-related tools that you have used in your program?
2. What does a successful/good transition to adult care look like for you?
3. What do you think is working well in your program to support the transition to adult care?
   1. How do you think your transition-focused services could be improved?
4. What are the barriers to helping youth and their families transition well?
   1. For those who do not focus on transition as their primary role: what are the barriers to focusing on transition?
5. When thinking about the transition support you provide (or that your clients receive), what is missing?
6. What do you think drives practice change to improve transition?
   1. What do providers need to improve transition?
   2. What knowledge do they need?
   3. What tools would help?
   4. What practice or policy changes are needed?
7. What is the role of community organizations and/or the education or social services in health care transition?
8. Is there anything that I missed or that you would like to add?
   1. Are there any important lessons that you have learned from delivering your transition program or service that you would like to share?
9. Do you know of other service providers that provide transition services or programs that might be interested in completing the survey on transition programs and services?

*Note: Additional questions may include questions to clarify or expand on participants’ responses to the environmental scan survey, which were collected prior to their enrolment in this study.
